# Adolescent Identity Search Algorithm Based on Fast Search and Balance Optimization for Numerical and Engineering Design Problems

**DOI:** 10.1155/2022/5692427

**Published:** 2022-08-01

**Authors:** Wentao Wang, Hao Liu, Quanqin He

**Affiliations:** School of Science, University of Science and Technology Liaoning, Anshan 114051, China

## Abstract

This paper proposed a fast convergence and balanced adolescent identity search algorithm (FCBAISA) for numerical and engineering design problems. The main contributions are as follows. Firstly, a hierarchical optimization strategy is proposed to balance the exploration and exploitation better. Secondly, a fast search strategy is proposed to avoid the local optimization and improve the accuracy of the algorithm; that is, the current optimal solution combines with the random disturbance of Brownian motion to guide other adolescents. Thirdly, the Chebyshev functional-link network (CFLN) is improved by recursive least squares estimation (RSLE), so as to find the optimal solution more effectively. Fourthly, the terminal bounce strategy is designed to avoid the algorithm falling into local optimization in the later stage of iteration. Fifthly, FCBAISA and comparison algorithms are tested by CEC2017 and CEC2022 benchmark functions, and the practical engineering problems are solved by algorithms above. The results show that FCBAISA is superior to other algorithms in all aspects and has high precision, fast convergence speed, and excellent performance.

## 1. Introduction

**Figure 1:**
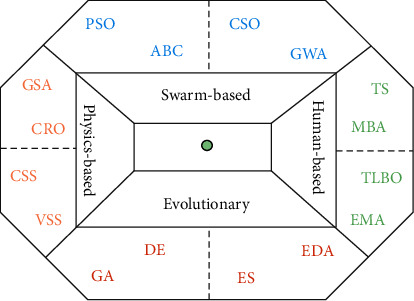
Classification of meta-heuristic techniques (meta-heuristic diamond).

Optimization is an important part to find better solutions when solving many scientific problems [1]. Many practical problems ultimately boil down to a set of decision variables that make the objective function to obtain the most optimal value. Researchers have found that meta-heuristic algorithms can solve many practical problems in the specified error range, which greatly improves the efficiency. Therefore, a variety of meta-heuristic algorithms are widely proposed by researchers, which are used to find approximate solutions of many complex problems. Based on studies from many researchers, meta-heuristic approaches can be divided into four main categories [2], and its details are shown in Figure 1.

A mature global optimization method with good stability and wide applicability is named evolutionary computation. EAs are inspired by the evolutionary operation of organisms in nature. They have characteristics of organization, adaptive, and learning, which can be applied to solve complex problems effectively, which is difficult to be solved by traditional optimization algorithms. Some of the renowned algorithms are genetic algorithm (GA) [3], differential evolution (DE) [4], estimation of distribution algorithms (EDA) [5], etc.

Swarm intelligence mainly simulates a group behavior of insects, herds, birds, and fish. Each member of the population constantly changes direction by learning its own experience and other members' experience. This phenomenon stimulates design algorithms and distributed problem solution. There are many such algorithms, for example, particle swarm optimization (PSO) [6], investigation of bee colony algorithm (ABC) [7], bacterial foraging algorithm (BFA) [8], Harris hawks optimization algorithm (HHO) [9], research on firefly algorithm (FA) [10], fruit fly optimization algorithm (FOA) [11], krill herd algorithm (KH) [12], research on crow search algorithm (CSA) [13], grass fibrous root optimization algorithm (FRO) [14], Flamingo search algorithm (FSA) [15], flow direction algorithm (FDA) [16], grey wolf optimizer (GWO) [17], battle royale optimization algorithm (BRO) [18] and coot swarm optimization (CSO) [19].

Meta-heuristic algorithms are based on physics or chemistry and formed by observing some physical or chemical phenomena and using their laws including gravity, potential energy, ecosystem, and motion. Simulated annealing (SA) [20], gravitational search algorithm (GSA) [21], noisy intermediate-scale quantum algorithms (NISQ) [22], chemical reaction optimization (CRO) [23], charged system search (CSS) [24], black hole (BH) [25], ions motion algorithm [26], multiverse optimizer (MVO) [27] and vortex search (VS) [26] are some optimizers in this category.

The last kind of meta-heuristic algorithm is based on human behavior, habit, thought, and logic. It is very popular in solving many problems, such as Tabu search (TS) [28], mine blast algorithm (MBA) [29], teaching–learning-based optimization (TLBO) [30], interior search algorithm (ISA) [31], exchange market algorithm (EMA) [32] and heuristic genetic algorithm (HGA) [33].

Optimization is applied to various real-life applications to reduce the waste of resources, save costs, reduce expenses, and maximize benefits. Researchers develop a large number of new algorithms or hybrid algorithms to solve real-life problems. In the process of product development, the newly developed political optimization algorithm (POA) was used and minimized the product cost, which is a new idea for industrial companies to fill the gap in their product design stage [34]. The new optimizer based on the ecogeography-based optimization algorithm (EBO) was applied to vehicle design for the first time, and better design results are obtained [35]. A new optimization algorithm based on grasshopper optimization algorithm and Nerdler–Mead algorithm (HGOANM) was developed to explore robot design of the robot gripper mechanism. The results showed that this algorithm can solve practical engineering problems quickly in Reference [36]. A new hybrid Taguchi salp swarm algorithm (HTSSA) was designed and used to speed up the optimization process of industrial structure design. The results reflected that the ability of HTSSA was superiority to optimize the product design process [37]. The new optimizer was developed, which is based on Seagull optimization (SOA), and its performance was verified by large-scale industrial engineering problems [38].

With the research and development of algorithms, the continuous development of optimization algorithm diversity is encouraged. A novel meta-heuristic approach based on human behavior for solving various complex optimization problems was introduced and called adolescent identity search algorithm (AISA) [39], which are first proposed by Esref Bogar and Selami Beyhan in 2020. This paper makes a series of improvements to AISA, which can make it performance better. The main contributions can be summarized as follows:

- This work divides the iteration into three layers and makes full use of the update mechanism of each layer to obtain the best adolescent identity, which can enrich population diversity, and balance the capabilities of exploration and exploitation better.
- The current optimal solution guides other adolescents to combine Brownian motion, which can accelerate the convergence speed of the algorithm and prevent the algorithm from local optimization.
- Recursive least squares estimation (RLSE) is proposed to estimate the weight factor better. Optimizing the improved CFLN can improve the ability of exploration and exploitation, which makes the optimal solution and can be found more effectively by the algorithm.
- To prevent AISA into local optimum at the late iteration, a terminal bounce strategy is proposed.

The structure of this paper is listed as follows. In Section 2, the adolescent identity search algorithm (AISA) is introduced.  Section 3 describes FCBAISA in detail. The experimental comparison among FCBAISA and other algorithms is presented and discussed in Section 4. The practical engineering problems are solved by FCBAISA and comparison algorithms in Section 5. In Section 6, the summaries of this paper and the future work based on FCBAISA are listed.

## 2. The Canonical AISA

An optimization algorithm constructed on human behavior was called AISA by Esref Bogar and Selami Beyhan in 2020. Through observing the formation process of adolescent identity and modeling it mathematically, a creative algorithm has been formed. This section briefly describes AISA, the details in Reference [39].

### 2.1. Population Random Initialization

In AISA, a random initial population is generated by

(1) $$\begin{array}{c} \begin{array}{c} x_{j}^{i}=lb_{j}+U{\left(0,1\right)}_{j}*\left(ub_{j}-lb_{j}\right),i=1,2,\ldots ,N;\\ j=1,2,\ldots ,n, \end{array} \end{array}$$

where *x*_*j*_^*i*^ is the *j*^*th*^ identity feature of the *i*^*th*^ adolescent and *U*(0,1) is an random number distributed uniformly in the range [0, 1]. *lb* is the lower boundary vectors of search space, and *ub* represents the upper.

### 2.2. Creating a New Identity

According to the characteristics of adolescent identity exploration, it is assumed that a situation is randomly selected during the iterative update. The three cases of adolescent identity feature selection in this algorithm are as follows:

**Case 1 .**

Teenagers form their identities by observing the surrounding society, judging social values, and choosing the correct beliefs and attitudes. Specifically, the Chebyshev functional-link network (CFLN) [40] approximation model is introduced to find the best adolescent identity, and the modeling process is as follows.

Chebyshev polynomials are shown in the following equation:

(2) $$\begin{array}{c} T_{s}\left(x\right)=\left\{\begin{array}{cc} 1, & \text{if }s=0,\\ x, & \text{if }s=1,\\ 2xT_{s-1}\left(x\right)-T_{s-2}\left(x\right), & \text{if }s\geq 2, \end{array}\right) \end{array}$$

where *s* is the degree of Chebyshev polynomials.

Normalizing input samples (population) for the CFLN model in [−1,1] by using the following equation:

(3) $$\begin{array}{c} {\hat{x}}_{j}^{i}=2\frac{x_{j}^{i}-lb_{j}}{ub_{j}-lb_{j}}-1, \end{array}$$

where ${\hat{x}}_{j}^{i}$ is normalized value of the *j*^*th*^ identity feature of the *i*^*th*^ adolescent. *lb* and *ub* are the lower and upper boundary vectors of search space. The identity is represented by the following normalized input matrix:

(4) $$\begin{array}{c} \hat{X}={\left[\begin{array}{cccc} {\hat{x}}_{1}^{1} & {\hat{x}}_{2}^{1} & \cdots & {\hat{x}}_{n}^{1}\\ {\hat{x}}_{1}^{2} & {\hat{x}}_{2}^{2} & \cdots & {\hat{x}}_{n}^{2}\\ \vdots & \vdots & \ddots & \vdots \\ {\hat{x}}_{1}^{N} & {\hat{x}}_{2}^{N} & \cdots & {\hat{x}}_{n}^{N} \end{array}\right]}_{N\times n}. \end{array}$$

Then, according to (2), the matrix Ψ of each input element is obtained by (5), and Ψ is the regression matrix.

(5) $$\begin{array}{c} \Psi ={\left[\begin{array}{ccccccc} T_{1}\left({\hat{x}}_{1}^{1}\right) & \cdots & T_{s}\left({\hat{x}}_{1}^{1}\right) & \cdots \cdots & T_{1}\left({\hat{x}}_{n}^{1}\right) & \cdots & T_{s}\left({\hat{x}}_{n}^{1}\right)\\ T_{1}\left({\hat{x}}_{1}^{2}\right) & \cdots & T_{s}\left({\hat{x}}_{1}^{2}\right) & \cdots \cdots & T_{1}\left({\hat{x}}_{n}^{2}\right) & \cdots & T_{s}\left({\hat{x}}_{n}^{2}\right)\\ \vdots & \ddots & \vdots & \ddots & \ddots & \vdots & \ddots \\ T_{1}\left({\hat{x}}_{1}^{N}\right) & \cdots & T_{s}\left({\hat{x}}_{1}^{N}\right) & \cdots \cdots & T_{1}\left({\hat{x}}_{n}^{N}\right) & \cdots & T_{s}\left({\hat{x}}_{n}^{N}\right) \end{array}\right]}_{N\times \left(n\times s\right)}\\ =\left[\begin{array}{ccc} \psi_{1}^{1} & \cdots \cdots & \psi_{n}^{1}\\ \psi_{1}^{2} & \cdots \cdots & \psi_{n}^{2}\\ \vdots & \ddots \ddots & \vdots \\ \psi_{1}^{N} & \cdots \cdots & \psi_{n}^{N} \end{array}\right]. \end{array}$$

Weighting factors are estimated by using the least square estimation (LSE) in approximate model as follows:

(6) $$\begin{array}{c} \hat{\omega }={\left(\Psi^{T}\Psi \right)}^{-1}\Psi^{T}f\\ \Psi =\left[\begin{array}{c} {\hat{\omega }}_{1}^{1},\ldots ,{\hat{\omega }}_{s}^{1},\cdots \;\cdots ,{\hat{\omega }}_{1}^{n},\ldots ,{\hat{\omega }}_{s}^{n} \end{array}\right]\\ ={\left[\begin{array}{c} \omega^{1},\cdots \;\cdots ,\omega^{n} \end{array}\right]}_{1\times \left(n\times s\right)}, \end{array}$$

where *ω*^*j*^ represents the weight vector of the *j*^*th*^ input.

All elements in (4) after normalization, the fitness values are calculated by (7) and stored in the matrix $\hat{F}$.

(7) $$\begin{array}{c} {\hat{f}}_{j}^{i}=\psi_{j}^{i}\omega^{i}, \end{array}$$

(8) $$\begin{array}{c} \hat{F}={\left[\begin{array}{cccc} {\hat{f}}_{1}^{1} & {\hat{f}}_{2}^{1} & \cdots & {\hat{f}}_{n}^{1}\\ {\hat{f}}_{1}^{2} & {\hat{f}}_{2}^{2} & \cdots & {\hat{f}}_{n}^{2}\\ \vdots & \vdots & \ddots & \vdots \\ {\hat{f}}_{1}^{N} & {\hat{f}}_{2}^{N} & \cdots & {\hat{f}}_{n}^{N} \end{array}\right]}_{N\times n}. \end{array}$$

Finally, the fitness values of the random initialization matrix elements are calculated and find the row index of the minimum value of each column in the matrix through the approximate model to form the best vector of identity of the present population, as shown in the following equation:

(9) $$\begin{array}{c} x_{j}^{*}=x_{j}^{m^{j}},m^{j}=\arg\min\left(l\right)\left\{{\hat{f}}_{j}^{l}|l=1,2,\ldots ,N\right\},\forall j. \end{array}$$

In Case 1, new identity of the *i*^*th*^ adolescent is defined as

(10) $$\begin{array}{c} x_{\text{new}}^{i}=x^{i}-r_{1}\left(x^{i}-x^{*}\right), \end{array}$$

where *r*_1_ ∈ [0,1] represents a random number, and *x*^*∗*^ represents the best identity feature created by each teenager in (8). The (10) represents a new identity that adolescents strive to acquire from their peer group with good behaviors.

**Case 2 .**

Believing that a role model has noble quality, good style, and imitating the role model to form the new identity.

Adolescents imitate the role model to form the new identity because they believe that a role model has noble quality and good style.

Therefore, adolescents can choose a better individual than themselves through learning. In this case, the updating formula for generate a new identity is written by the following equation:

(11) $$\begin{array}{c} x_{\text{new}}^{i}=x^{i}-r_{2}\left(x^{p}-x^{rm}\right), \end{array}$$

where *r*_2_ ∈ [0,1] is a random number, and *x*^*rm*^ is the role model, which the best individual. When *p* ≠ *rm*, *x*^*p*^ is an adolescent selected in the population randomly.

**Case 3 .**

Adolescents may be negatively affected by the group and form bad identity choices such as smoking, dropping out of school, and fighting. In this case, the updating formula for obtaining the new identity of the *i*^*th*^ adolescent is written by the following equation:

(12) $$\begin{array}{c} x_{\text{new}}^{i}=x^{i}-r_{3}\left(x^{i}-x^{q}\right), \end{array}$$

where *r*_3_ ∈ [0,1] is an *n*-dimensional vector of uniformly distributed numbers in the interval [0, 1], and *x*^*q*^ is a negative identity vector and is written by the following equation:

(13) $$\begin{array}{c} x^{q}={\left[\begin{array}{c} x^{u},x^{u},\ldots ,x^{u} \end{array}\right]}_{1\times n}^{T}, \end{array}$$

where *x*^*u*^ is negative identity feature, which is an element randomly selected from the population matrix to make the algorithm that has the exploration capability.

## 3. The Proposed FCBAISA

Different from other meta-heuristic algorithms, AISA tries to find the fitness of adolescents and uses CFLN optimization. AISA performance is good in exploration, exploitation, avoidance of local optimization, and convergence. However, there are also some problems such as unbalanced exploration and exploitation abilities, falling into local optimum, and premature convergence. Adolescent identity development is a complex concept, which can integrate different network structures. Therefore, new ideas can still be injected into the algorithm.

### 3.1. Hierarchical Optimization Strategy

**Figure 2:**
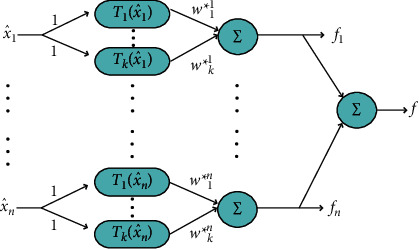
Topology of improved CFLN.

CFLN optimization method is very novel and effective for exploration, which is used in Case 1. In order to better play the role of CFLN, the iteration is divided into three layers to execute each update mechanism separately in this paper. This strategy can increase the diversity of the population and balance the abilities of exploration and exploitation better. In addition, improved CFLN topology in Section 3.3 has the better ability of exploration, as shown in Figure 2.

### 3.2. Quick Search Strategy

**Figure 3:**
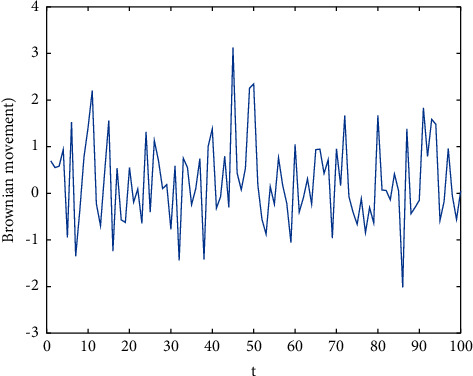
Brownian motion.

This paper uses the current optimal solution (*G*best) to guide other adolescents in the whole search process and uses the characteristic that Brownian motion obeys standard normal distribution to design a fast search strategy to speed up the convergence speed of the algorithm. The *G*best guides other adolescents to update. In most cases, the optimal solution can be found faster. In addition, Brownian motion [41] is introduced to form a new update mechanism, because Brownian motion can replace random disturbance and effectively accelerate the convergence speed of the algorithm. This method enables teenagers to obtain the best adolescent identity as soon as possible, as shown in Figure 3.

Based on the current optimal solution (*G*best) and Brownian motion, as shown in (14), the formula of Case 1 is changed to the following equation.

(14) $$\begin{array}{c} x_{pb}^{i}=b_{1}.*\left(G\text{best}-x^{i}\right), \end{array}$$

(15) $$\begin{array}{c} x_{\text{new}}^{i}=x^{i}-b_{2}*\left(r_{1}*x^{i}-x^{*}\right)-b_{3}*x_{pb}, \end{array}$$

where *b*_1_ is *n*-dimensional Brownian motion. *r*_1_ ∈ [0,1] represents a random number, and *b*_2_ and *b*_3_ are two random numbers generated by Brownian motion.

In addition, Brownian motion is integrated into Cases 2 and 3, and the corresponding update formulates are changed as (16) and (17), respectively.

(16) $$\begin{array}{c} x_{\text{new}}^{i}=x^{i}-\text{rand}n*\left(x^{p}-x^{rm}\right), \end{array}$$

(17) $$\begin{array}{c} x_{\text{new}}^{i}=x^{i}-\text{rand}n*\left(x^{i}-x^{q}\right). \end{array}$$

### 3.3. RLSE Weight Factor Strategy

The classical least square estimator (LSE) can be written as follows:

(18) $$\begin{array}{c} A_{0}X_{0}=b_{0}, \end{array}$$

(19) $$\begin{array}{c} \left[\begin{array}{c} A_{0}\\ A_{1} \end{array}\right]X_{1}=\left[\begin{array}{c} b_{0}\\ b_{1} \end{array}\right], \end{array}$$

where *A*_0_ is a *N* × *n* matrix, *X*_0_=[*X*_1_, *X*_2_,…,*X*_*n*_]^*T*^ is a *n* × 1 parameter vector, and *b*_0_=[*b*_1_, *b*_2_,…,*b*_*N*_]^*T*^ is an output vector. The LSE can be given from following equations:

(20) $$\begin{array}{c} X_{0}={\left(A_{0}^{T}A_{0}\right)}^{-1}A_{0}^{T}b_{0}, \end{array}$$

(21) $$\begin{array}{c} X_{1}=\left({\left[\begin{array}{c} A_{0}\\ A_{1} \end{array}\right]}^{T}{\left[\begin{array}{c} A_{0}\\ A_{1} \end{array}\right]}^{-1}\right){\left[\begin{array}{c} A_{0}\\ A_{1} \end{array}\right]}^{T}\left[\begin{array}{c} b_{0}\\ b_{1} \end{array}\right]. \end{array}$$

In AISA, the weight factor is estimated by (19), which is the classical LSE recursive least squares estimation (RSLE) [42] and is used to optimize the least square estimation and estimate the weigh factor of its approximate model.

(22) $$\begin{array}{c} G_{1}={\left[\begin{array}{c} A_{0}\\ A_{1} \end{array}\right]}^{T}\left[\begin{array}{c} A_{0}\\ A_{1} \end{array}\right]=G_{0}+A_{1}^{T}A_{1},\\ {\left[\begin{array}{c} A_{0}\\ A_{1} \end{array}\right]}^{T}{\left[\begin{array}{c} b_{0}\\ b_{1} \end{array}\right]}^{-1}=G_{1}X_{0}+\left(b_{1}-A_{1}X_{0}\right)A_{1}^{T},\\ \begin{array}{c} X_{1}=G_{1}^{-1}G_{1}X_{0}+G_{1}^{-1}A_{1}^{T}\left(b_{1}-A_{1}X_{0}\right)\\ =X_{0}+G_{1}^{-1}A_{1}^{T}\left(b_{1}-A_{1}X_{0}\right) \end{array},\\ X_{s+1}=X_{s}+G_{s+1}^{-1}A_{s+1}^{T}\left(b_{s+1}-A_{s+1}X_{s}\right), \end{array}$$

where we eliminate *A*_0_ and *b*_0_ variables, *G*_0_=*A*_0_^*T*^*A*_0_, *X*_0_=*G*_0_^−1^*A*_0_^*T*^*b*_0_.

In AISA, CFLN uses the LSE to estimate the weight factor of the approximate model. In this paper, a dynamic way to estimate the weight factor of the approximate model based on the LSE by learning from the recursive proof of RLSE. RLSE can dynamically estimate the weight factor of the approximate model and make CFLN more efficient as shown in Figure 2. FCBAISA can find the optimal solution more efficient by modifying the approximate model to affect the algorithm update mechanism. The formula is changed as (27).

(23) $$\begin{array}{c} \omega ={\left(\Psi^{T}\Psi \right)}^{-1}\Psi^{T}f, \end{array}$$

(24) $$\begin{array}{c} \omega^{*}=\omega +{\left(\Psi^{T}\Psi \right)}^{-1}\Psi^{T}\left(f-\Psi \omega \right), \end{array}$$

(25) $$\begin{array}{c} \begin{array}{c} \hat{\omega }={\left[\begin{array}{ccccccc} {\hat{\omega }}_{1}^{1} & \cdots & {\hat{\omega }}_{s}^{1} & \cdots \;\cdots & {\hat{\omega }}_{1}^{n} & \cdots & {\hat{\omega }}_{s}^{n} \end{array}\right]}_{1\times \left(n\times s\right)}\\ ={\left[\begin{array}{ccc} \omega^{1} & \cdots \;\cdots & \omega^{n} \end{array}\right]}_{1\times \left(n\times s\right)} \end{array}. \end{array}$$

### 3.4. Terminal Bounce Mechanism

In this paper, a terminal bounce mechanism is designed to avoid the algorithm falling into local optimization in the later stage of iteration. Specifically, the algorithm may fall into local optimization if the number of iterations increases, especially in the later stage of iteration, while the value of global optimization does not change within the specified number of iterations. In this paper, the value of the timer is set to 20 and adds a counter to monitor the change of the global optimum value, which is the end disturbance mechanism that will be triggered when there is no change in the global optimum value after 20 iterations, which can make the algorithm jump out of the local optimum. In order to achieve better disturbance effect, two individuals are selected randomly from the population and the *G*best is added for guidance when designing the end disturbance strategy. The pseudocode is given by Algorithm 1, and the specific design of the terminal bounce mechanism is as formula (26).

(26) $$\begin{array}{c} x=r*G\text{best}+\left(1-r\right)*\left(\text{rand}*\left(x\left(ind\left(1\right)\right)-x\left(ind\left(2\right)\right)\right)\right), \end{array}$$

where *G*best represents the current optimal solution, *r* ∈ [0,1] denotes a random number, and *ind*(1) and *ind*(2) are two indexes generated from the population randomly.

**Figure 4:**
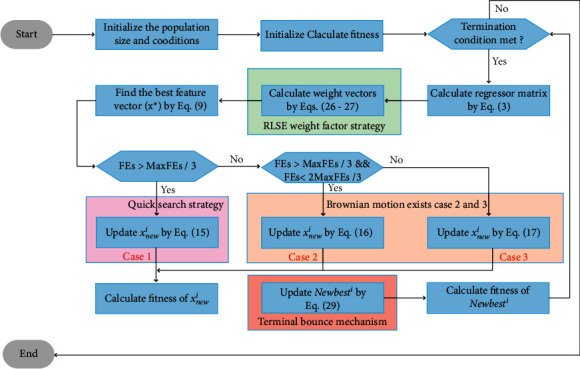
Flowchart of FCBAISA.

In summary, a fast convergence and balanced AISA is proposed (FCBAISA), Algorithm 2, and Figure 4 gives the pseudocode and flowchart of FCBAISA, respectively.

## 4. Experimental Results and Analysis

### 4.1. Benchmark Function and Comparison Algorithm

The CEC2017 benchmark functions are applied to check the performance of FCBAISA in this paper. Among the CEC2017 benchmark functions [43], {*f*_1_, *f*_3_}, {*f*_4_ ~ *f*_10_}, {*f*_11_ ~ *f*_20_}, and {*f*_21_ ~ *f*_30_} are unimodal functions, simple multimodal functions, hybrid functions, and composite functions, respectively. *f*_2_ has not been tested, the reason is the instability in high dimensional, and the details can be found in Reference [44]. The CEC2022 benchmark function includes unimodal function, basic functions, hybrid function, and composition function. These benchmark functions are detailed in Tables 1 and 2.

For checking the effectiveness and superiority of FCBAISA, it is compared with the performance of eight evolutionary algorithms. In order to be more fair and reasonable, the comparison algorithms include the classical algorithm and the new excellent algorithm. These are as follows: transient search algorithm (TSO) [45], the Archerfish Hunting Optimizer algorithm (AHO) [46], butterfly optimization algorithm (BOA) [47], dynamic differential annealed optimization (DDAO) [48], PSO [6], owl search algorithm (OSA) [49], and gravitational search algorithm (GSA) [50]. The contents of these algorithms are shown in Table 3. To compare the performance of algorithms fairly, the population size (*N*) of all algorithms is 30, the dimension (*n*) is 30, and each algorithm runs 50 times independently. The maximum number of function evaluations is 30000, and the maximum number of iterations is 1000 in the CEC2017 benchmark functions. The population size (*N*) of all algorithms is 30, the dimension (*n*) is 20, and each algorithm runs 50 times independently. The maximum number of function evaluations is 100000, and the maximum number of iterations is 3334 in the CEC2022 benchmark functions.

### 4.2. Comparison between FCBAISA and Other Algorithms

In order to be more fair and reasonable, the comparison algorithm includes the classical algorithm and the new excellent algorithm. The results of CEC2017 and CEC2022 benchmark functions are shown in Tables 4 and 5, respectively. Among the CEC2017 benchmark functions, FCBAISA ranks first in 21, second in 6, and first after the comprehensive comparison. For other algorithms, the comprehensive performance of PSO is better, ranking third. From the mean comparison, it is found that FCBAISA performs better on 21 benchmark functions, and GSA and PSO perform better on four and three test functions, respectively. From the comparison of standard deviation, it is found that the stability of FCBAISA is poor, but it also ranks first in 15 benchmark functions. In complex problems, the stability of FCBAISA is improved. By comparing the optimal solutions of each algorithm, FCBAISA can find a better optimal solution among 16 benchmark functions in CEC2017 benchmark functions. In CEC2022 benchmark functions, FCBAISA ranks first in 10 benchmark functions, second in 2 benchmark functions, and first after the comprehensive comparison. From the mean comparison, it is found that FCBAISA performs better on 10 benchmark functions. It is concluded that the FCBAISA algorithm can effectively solve the simple and complex problems, especially when solving complex problems, it is better than other algorithms. In general, FCBAISA performs better in all aspects and can find the optimal solution quickly and efficiently in most benchmark functions.

### 4.3. Convergence Rate

**Figure 5:**
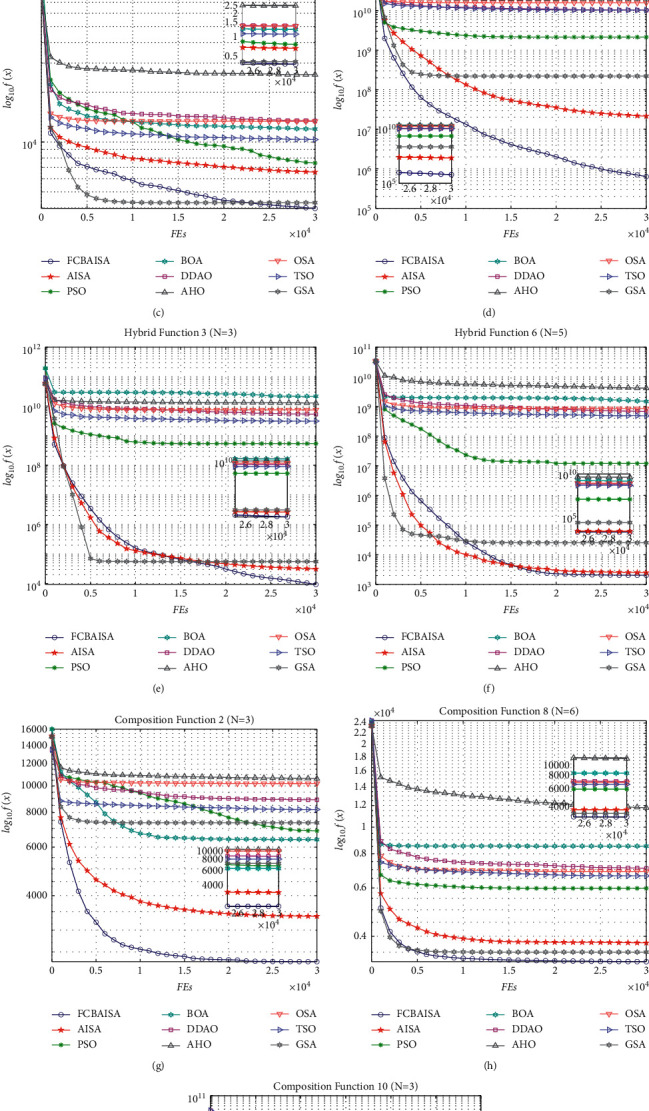
Convergence curves of 9 algorithms in CEC2017 benchmark functions. (a) *f*_1_. (b) *f*_3_. (c) *f*_9_. (d) *f*_12_. (e) *f*_13_. (f) *f*_19_. (g) *f*_22_. (h) *f*_28_. (i) *f*_30_.

**Figure 6:**
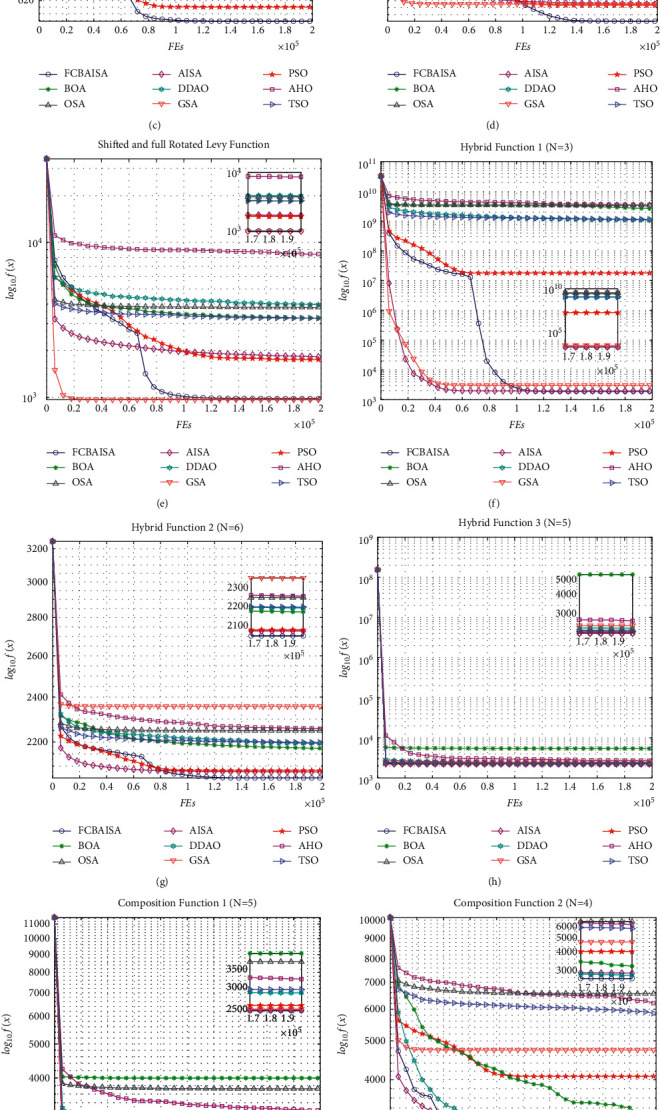
Convergence curves of 9 algorithms in CEC2022 benchmark functions. (a) *f*_1_. (b) *f*_2_. (c) *f*_3_. (d) *f*_4_. (e) *f*_5_. (f) *f*_6_. (g) *f*_7_. (h) *f*_8_. (i) *f*_9_. (j) *f*_10_. (k) *f*_11_. (l) *f*_12_.

In CEC2017 benchmark functions, *f*_1_ and *f*_3_ are two unimodal functions, and FCBAISA has a very fast convergence rate. *f*_9_is a simple multimodal function. At the beginning of the iteration, the decline speed of GSA in the convergence curve is faster than that of FCBAISA. But in the later stage of the iteration, FCBAISA exceeds GSA, indicating the strong development ability of the improved algorithm. *f*_12_, *f*_13_ and *f*_19_ are hybrid functions. The faster the convergence speed of FCBAISA, the greater the advantages of FCBAISA in this kind of functions. *f*_22_, *f*_28_, and *f*_30_ are composite functions, and the convergence speed of FCBAISA is the fastest among the three composite functions and indicates that FCBAISA has strong performance in solving complex problems. The convergence curve is shown in Figure 5. In CEC2022 benchmark functions, FCBAISA performs better on most benchmark functions, and all details are in Figure 6.

### 4.4. Statistical Analysis

For testing the FCBAISA and the above experimental results, statistical analysis is carried out, including the Wilcoxon rank test, Friedman test, and Quade test. Wilcoxon rank test mainly checks the performance of FCBAISA and compares algorithms one by one. Friedman test and Quaid test mainly test all algorithms together, then compare the performance of the algorithm from the overall point of view, and finally give the ranking and *p*_value. Through these tests, the performance of the improved algorithm can be well tested.

For the Wilcoxon rank test, its criterion is when the significance level is 0.05, when *p*_value ≤ 0.05, if *R*^+^ < *R*^−^ is marked as “+,” FCBAISA and other algorithms are significantly better. On the contrary, it will be marked as “+,” indicating that FCBAISA performs worse than other algorithms. If there is no significant difference between FCBAISA and other algorithms, it will be marked as “=.” In Tables 6 and 7, the last row gives the sum of each tag to judge whether FCBAISA has significant advantages over other comparison algorithms. In comparison with other improved algorithms, FCBAISA got “+” on at least 25 benchmark functions, except for 20 “+” compared with PSO, and only two “−.” To sum up, these tests show that the performance of FCBAISA is better than other comparing algorithms.

For Friedman and Quade test, the significance level is 0.05, if *p*_value≤ 0.05, indicating that the test result is true. The results of the Friedman and Quade tests are shown in Tables 8 and 9 and indicate that the FCBAISA ranks first. After the Friedman test result in Table 8, the FCBAISA has a *p*_value of 1.2942E-10 and the test result is 8.6552. For Quade test result in Table 9, it can be observed that FCBAISA's final ranking is number one. FCBAISA's result is 8.8229 with a *p*_value of 2.2054E-43 in the Quade test. In summary, after three statistical tests, it can be proved that FCBAISA has significant advantages over 8 other comparative algorithms, including improved algorithms and other excellent algorithms.

## 5. Practical Engineering Problems

This section uses FCBAISA and all comparison algorithms to solve several problems of engineering design. The superiority of FCBAISA is further tested by analyzing those experimental results. Engineering design problems include pressure vessel design [51], welded beam design [52], gear train engineering design [53, 54], and speed reducer design, and the details are as follows.

### 5.1. The Problem of Pressure Vessel Design

**Figure 7:**
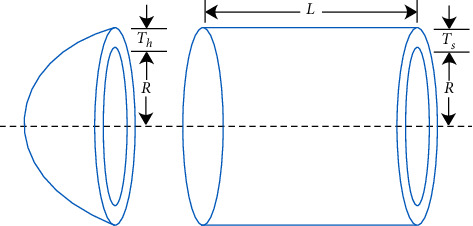
Model design.

Pressure vessels are designed to minimize costs. The pressure vessel, as shown in Figure 7, consists of a cylindrical center and hemispherical heads at both ends, where *L* (*x*_4_), *T*_*s*_ (*x*_1_), *T*_*h*_ (*x*_2_), and *R*(*x*_3_) are the length of the cylindrical part, the thickness of the shell, the thickness of head, and the inner radius, respectively. This problem consists of four constraints, including three linear inequalities and a nonlinear inequality, and its model is shown in the following equation.

(27) $$\begin{array}{c} \text{Min }f\left(x\right)=0.6224x_{1}x_{3}x_{4}+1.7781x_{2}x_{3}^{2}\\ +3.1611x_{1}^{2}x_{4}+19.84x_{1}^{2}x_{3,}\\ s.t.\left\{\begin{array}{c} y_{1}\left(x\right)=-x_{1}+0.0193x_{3}\leq 0,\\ y_{2}\left(x\right)=-x_{2}+0.00954x_{3}\leq 0,\\ y_{3}\left(x\right)=-\pi x_{3}^{2}x_{4}-\frac{4}{3}\pi x_{3}^{3}+1,296,000\leq 0,\\ y_{4}\left(x\right)=x_{4}-240\leq 0.\\ 1\leq x_{1},x_{2}\leq 99,\;10\leq x_{3},x_{4}\leq 200. \end{array}\right) \end{array}$$

**Figure 8:**
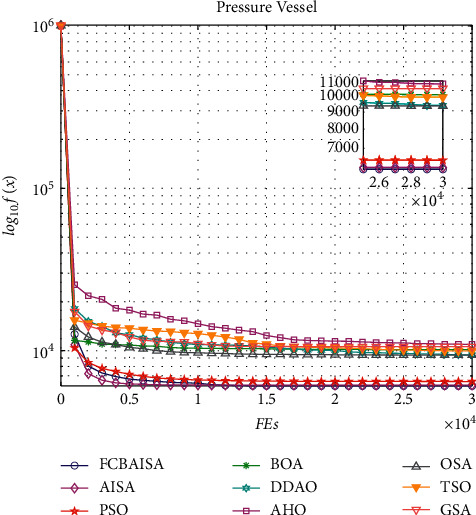
Convergence curves of 9 algorithms in pressure vessel design.

In Table 10, it can be seen that the optimal solution of each algorithm in solving the problem of pressure vessel design is 6.06E-10, and FCBAISA is better than that of other algorithms. The convergence curve of the algorithm involved in this paper on pressure vessel problem is shown in Figure 8.

### 5.2. The Problem of Welded Beam Design

**Figure 9:**
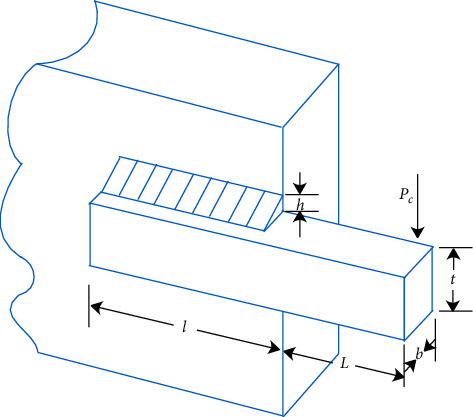
Model design.

In this design problem, the main restrict factors of the design cost of welded beams include shear stress (*τ*), bending stress (*σ*) in the beam, buckling load on the bar (*P*_*c*_), end deflection of the beam (*δ*), and side constraints. The variables involved including *h*(*x*_1_), *l*(*x*_2_), *t*(*x*_3_), and *b*(*x*_4_) in this design problem, and the details are shown in Figure 9. This problem consists of seven constraints, including two linear and five nonlinear inequality, its model as in equation (28), where *x* = [*x*_1_, *x*_2_, *x*_3_, *x*_4_] = [*h*, *l*, *t*, *b*].

(28) $$\begin{array}{c} \text{Min }f\left(x\right)=1.10471x_{1}^{2}x_{2}+0.04811x_{3}x_{4}\left(14+x_{2}\right),\\ s.t.\left\{\begin{array}{c} g_{1}\left(x\right)=\tau \left(x\right)+\tau_{\max}\leq 0,\\ g_{2}\left(x\right)=\sigma \left(x\right)+\sigma_{\max}\leq 0,\\ g_{3}\left(x\right)=\delta \left(x\right)+\delta_{\max}\leq 0,\\ g_{4}\left(x\right)=x_{1}-x_{4}\leq 0,\\ g_{5}\left(x\right)=P-P_{c}\left(x\right)\leq 0,\\ g_{6}\left(x\right)=0.125-x_{1}\leq 0,\\ g_{7}\left(x\right)=0.10471x_{1}^{2}+0.04811x_{3}x_{4}\left(14+x_{2}\right)\\ -5\leq 0,\\ 0.1\leq x_{1},x_{4}\leq 2,\;0.1\leq x_{2},x_{3}\leq 10, \end{array}\right) \end{array}$$

where $\begin{array}{c} \tau \left(x\right)=\sqrt{{\left(\tau^{\prime }\right)}^{2}+2\tau^{\prime }\tau^{\prime \prime }x_{2}/2R+{\left(\tau^{\prime \prime }\right)}^{2}},\tau^{\prime }=P/\sqrt{2}x_{1}x_{2},\tau^{\prime \prime }=MR/JM=P\left(L+x_{2}/2\right),\;R=\sqrt{x_{2}^{2}/4+{\left(x_{1}+x_{3}/2\right)}^{2}},\\ J=2\left\{\sqrt{2}x_{1}x_{2}\left[x_{2}^{3}/4+{\left(x_{1}+x_{3}/2\right)}^{2}\right]\right\}\sigma \left(x\right)=6PL/x_{4}x_{3}^{2},\delta \left(x\right)=4PL^{3}/Ex_{4}x_{3}^{3},P_{c}\left(x\right)=4.013E\sqrt{x_{3}^{2}x_{4}^{6}/36}/L^{2}\left\{1-x_{3}/2L\sqrt{E/4G}\right\} \end{array}$ and where *P* = 6000*lb*, *L* = 14*in*, *E* = 30 × 10^6^*psi*, *G* = 12 × 10^6^*psi*, *τ*_max_ = 13600*psi*, *σ*_max_ = 30000*psi*, *δ*_max_ = 0.25*in*.

**Figure 10:**
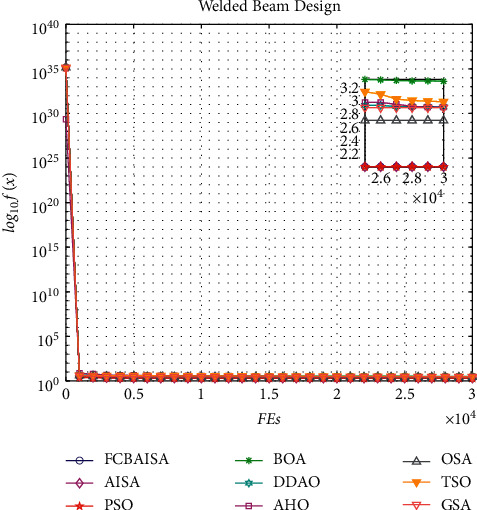
Convergence curves of 9 algorithms in welded beam design problem.

In Table 11, it can be seen that the optimal solution of each algorithm in solving the problem of welded beam design is 2.0632, and FCBAISA is better than that of other algorithms. The convergence curve of the algorithm involved in this paper on welded beam design problem is shown in Figure 10.

### 5.3. The Problem of Gear Train Engineering Design

**Figure 11:**
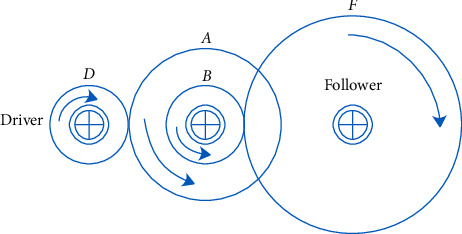
Model design.

The problem of gear train engineering design is to find the minimum value of gear and tooth ratio without affecting the efficiency as shown in Figure 11. The number of teeth must be an integer; thus, the design variables for this problem are discrete. Because constraints are constraints on design variables, the problem of constraints on discrete variables can increase its complexity. So, in this design problem, *n*_*A*_, *n*_*B*_, *n*_*D*_, and *n*_*F*_ are decision variables, the integer variable of the upper bound is 60, and the lower is 12. Besides, the gear ratio is defined as (*n*_*B*_*n*_*D*_)/(*n*_*F*_*n*_*A*_), this specific problem can be modelled as (29), where *x*=[*x*_1_, *x*_2_, *x*_3_, *x*_4_]=[*n*_*A*_, *n*_*B*_, *n*_*D*_, *n*_*F*_].

(29) $$\begin{array}{c} \text{Min }f\left(x\right)={\left(\frac{1}{6.931}-\frac{x_{1}x_{2}}{x_{3}x_{4}}\right)}^{2}.\\ s.t.12\leq x_{i}\leq 60,x_{i}\in N,i=1,2,3,4. \end{array}$$

**Figure 12:**
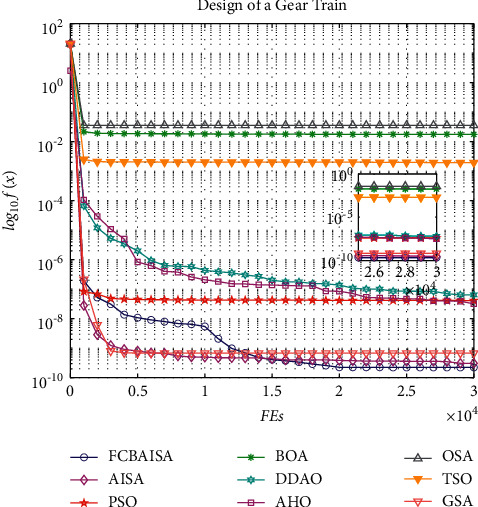
Convergence curves of 9 algorithms in gear train design problem.

In Table 12, it can be seen that the optimal solution of each algorithm in solving the problem of gear train engineering design is 2.23E-10, and FCBAISA is also better than that of other algorithms. The convergence curve of the algorithm involved in this paper on gear train engineering design problem is shown in Figure 12.

### 5.4. The Problem of Speed Reducer Design

**Figure 13:**
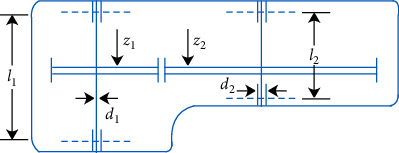
Model design.

In this constrained optimization problem (see Figure 13), the variables *x*_1_, *x*_2_, and *x*_3_, are face width (*b*), teeth module (*m*), teeth number (*z*), and *x*_4_, *x*_5_, and *x*_6_ represent length of the first shaft (*l*_1_), the second length (*l*_2_), and the diameter between *l*_1_ and *l*_2_(*d*_2_). This problem consists of 4 linear and 7 nonlinear inequalities, and the model of this specific problem is as (30), where *x*=[*x*_1_, *x*_2_, *x*_3_, *x*_4_, *x*_5_, *x*_6_, *x*_7_]=[*b*, *m*, *z*, *l*_1_, *l*_2_, *d*_1_, *d*_2_].

(30) $$\begin{array}{c} \text{Min }f\left(x\right)=0.7854x_{1}x_{2}^{2}\left(3.3333x_{3}^{2}+14.9334x_{3}-43.0934\right)-1.508x_{1}\left(x_{6}^{2}+x_{7}^{2}\right)+7.4777\left(x_{6}^{3}+x_{7}^{3}\right)+0.7854\left(x_{4}x_{6}^{2}+x_{5}x_{7}^{2}\right),\\ \begin{array}{c} s.t.\left\{\begin{array}{c} y_{1}\left(x\right)=\frac{27.0}{x_{1}x_{2}^{2}x_{3}}-1\leq 0,\\ y_{2}\left(x\right)=\frac{397.50}{x_{1}x_{2}^{2}x_{3}^{2}}-1\leq 0,\\ y_{3}\left(x\right)=\frac{1.930x_{4}^{2}}{x_{2}x_{6}^{4}x_{3}}-1\leq 0,\\ y_{4}\left(x\right)=\frac{1.930x_{5}^{2}}{x_{2}x_{7}^{4}x_{3}}-1\leq 0,\\ y_{5}\left(x\right)=\frac{\sqrt{{\left(745.0x_{4}/x_{2}x_{3}\right)}^{2}+16.90\times 10^{6}}}{110.0x_{6}^{3}}-1\leq 0,\\ y_{6}\left(x\right)=\frac{\sqrt{{\left(745.0x_{5}/x_{2}x_{3}\right)}^{2}+157.50\times 10^{6}}}{85.0x_{7}^{3}}-1\leq 0,\\ y_{7}\left(x\right)=\frac{x_{2}x_{3}}{40}-1\leq 0,\\ y_{8}\left(x\right)=\frac{5x_{2}}{x_{1}}-1\leq 0,\\ y_{9}\left(x\right)=\frac{x_{1}}{12x_{2}}-1\leq 0,\\ y_{10}\left(x\right)=\frac{1.50x_{6}+1.90}{x_{4}}-1\leq 0,\\ y_{11}\left(x\right)=\frac{1.10x7+1.90}{x_{5}}-1\leq 0,\\ 2.60\leq x_{1}\leq 3.60,\;0.70\leq x_{2}\leq 0.80,\\ 17.0\leq x_{3}\leq 28.0,\\ \;7.30\leq x_{4}\leq 8.30\\ 7.50\leq x_{5}\leq 8.30,\;2.90\leq x_{6}\leq 3.90,\\ 5.0\leq x_{7}\leq 5.50. \end{array}\right) \end{array} \end{array}$$

**Figure 14:**
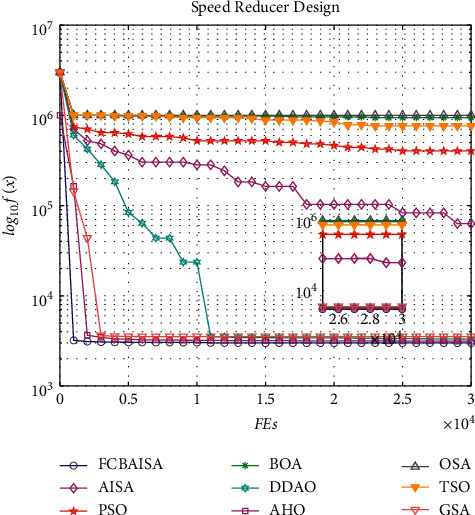
Convergence curves of 9 algorithms in speed reducer design problem.

In Table 13, it can be seen that the optimal solution of each algorithm in solving the problem of speed reducer design is 3.00E + 03, and FCBAISA is better than that of other algorithms obviously. The convergence curve of the algorithm involved in this paper on speed reducer design problem is shown in Figure 14.

## 6. Conclusion

A fast convergence and balanced adolescent identity search algorithm (FCBAISA) is proposed in this work for numerical and engineering design problems to advance the quality of AISA. To balance the exploration and exploitation of FCBAISA better, a layered optimization strategy is proposed. A fast search strategy is proposed to make the algorithm break away from the local optimization and converge to the optimal value faster. The CFLN is improved by RSLE to obtain the optimal result effectively. A terminal disturbance strategy is designed to prevent the algorithm from local optimization in the later iteration. The CEC2017 benchmark functions, CEC2022 benchmark functions, and the design problems of engineering are applied to check the quality of FCBAISA. It is clear that FCBAISA has high precision, fast convergence speed, strong exploration, and exploitation ability, and the balance between them is better. In addition, future research can be carried out from the following aspects:

1. Further improvement of FCBAISA, including the Chebyshev approximation model and other effective alternative models.
2. Trying to apply FCBAISA to the problems of multi-objective optimization, and considering the combination of specific practical problems, including scheduling optimization and engineering problems.

## Figures and Tables

**Algorithm 1:**
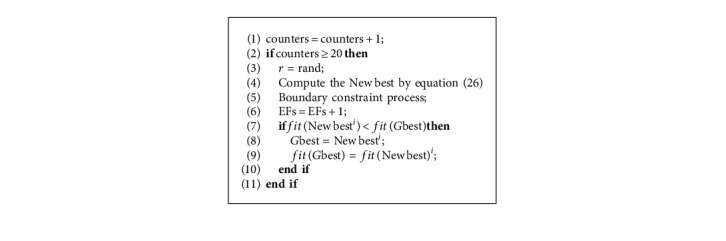
Terminal bounce mechanism.

**Algorithm 2:**
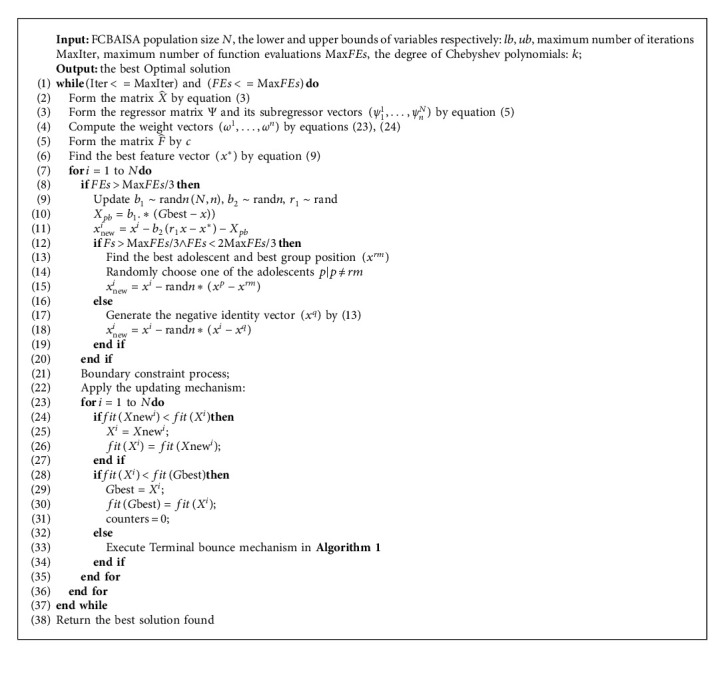
Pseudocode of FCBAISA.

**Table 1:** The information of the CEC2022 benchmark functions used in this paper.

	No.	Functions	*f*_opt_
Unimodal function	1	Shifted and full-rotated Zakharov function	300

Basic functions	2	Shifted and full-rotated Rosenbrock's function	400
	3	Shifted and full-rotated expanded Schaffer's f6 function	600
	4	Shifted and full-rotated noncontinuous Rastrigin's function	800
	5	Shifted and full-rotated levy function	900

Hybrid functions	6	Hybrid function 1 (*N* = 3)	1800
	7	Hybrid function 2 (*N* = 6)	2000
	8	Hybrid function 3 (*N* = 5)	2200

Composition functions	9	Composition function 1 (*N* = 5)	2300
	10	Composition function 2 (*N* = 4)	2400
	11	Composition function 4 (*N* = 6)	2600
	12	Composition function 4 (*N* = 6)	2700

Search range: [−100,100]			

**Table 2:** The information of the CEC2017 benchmark functions used in this paper.

Fun	Function	Range	*f*_*opt*_
*f*_1_	Shifted and rotated bent cigar function	[−100,100]	100
*f*_3_	Shifted and rotated Zakharov function	[−100,100]	300
*f*_4_	Shifted and rotated Rosenbrock's function	[−100,100]	400
*f*_5_	Shifted and rotated Rastrigin's function	[−100,100]	500
*f*_6_	Shifted and rotated expanded Scaffer's function	[−100,100]	600
*f*_7_	Shifted and rotated Lunacek bi-Rastrigin function	[−100,100]	700
*f*_8_	Shifted and rotated noncontinuous Rastrigin's function	[−100,100]	800
*f*_9_	Shifted and rotated levy function	[−100,100]	900
*f*_10_	Shifted and rotated Schwefel's function	[−100,100]	1000
*f*_11_	Hybrid function 1 (*N* = 3)	[−100,100]	1100
*f*_12_	Hybrid function 2 (*N* = 3)	[−100,100]	1200
*f*_13_	Hybrid function 3 (*N* = 3)	[−100,100]	1300
*f*_14_	Hybrid function 4 (*N* = 4)	[−100,100]	1400
*f*_15_	Hybrid function 5 (*N* = 4)	[−100,100]	1500
*f*_16_	Hybrid function 6 (*N* = 4)	[−100,100]	1600
*f*_17_	Hybrid function 6 (*N* = 5)	[−100,100]	1700
*f*_18_	Hybrid function 6 (*N* = 5)	[−100,100]	1800
*f*_19_	Hybrid function 6 (*N* = 5)	[−100,100]	1900
*f*_20_	Hybrid function 6 (*N* = 6)	[−100,100]	2000
*f*_21_	Composition function 1 (*N* = 3)	[−100,100]	2100
*f*_22_	Composition function 2 (*N* = 3)	[−100,100]	2200
*f*_23_	Composition function 3 (*N* = 4)	[−100,100]	2300
*f*_24_	Composition function 4 (*N* = 4)	[−100,100]	2400
*f*_25_	Composition function 5 (*N* = 5)	[−100,100]	2500
*f*_26_	Composition function 6 (*N* = 5)	[−100,100]	2600
*f*_27_	Composition function 7 (*N* = 6)	[−100,100]	2700
*f*_28_	Composition function 8 (*N* = 6)	[−100,100]	2800
*f*_29_	Composition function 9 (*N* = 3)	[−100,100]	2900
*f*_30_	Composition function 10 (*N* = 3)	[−100,100]	3000

**Table 3:** Relevant parameter values of the algorithm.

Algorithm	Years	Parameter information	Values
AISA [39]	2020	Number of Chebyshev polynomials (s)	3
TSO [45]	2020	NScaling factor	0.85
AHO [46]	2021	Theta	pi/12
		Omega	0.01
		maxCount	10

BOA [47]	2019	*p*	0.8
		*a*	0.1
		*c*	0.01

DDAO [48]	2020	MaxSubIt	10
		*T*_0_	2000
		Alpha	0.995

PSO [6]	1998	*c*_1_, *c*_2_	2
		*w*	0.9–0.4

OSA [49]	2018	Beta	0–1.9
		Epsilon	le-16

GSA [50]	2009	Rnorm	2
		ElitistCheck	1
		Minflag	1

FCBAISA presented	2021	Number of Chebyshev polynomials (s)	30

**Table 4:** Experimental results of FCBAISA and other algorithms in CEC2017 benchmark functions.

Function		TSO	AHO	BOA	DDAO	PSO	OSA	GSA	AISA	FCBAISA
*f*_1_	Mean	5.04E + 10	1.19E + 11	6.05E + 10	5.20E + 10	1.73E + 10	5.68E + 10	4.50E + 06	5.14E + 09	9.23E + 03
	Std	7.51E + 09	1.44E + 10	3.99E + 09	4.89E + 09	8.06E + 09	6.01E + 09	1.65E + 07	3.25E + 09	2.35E + 04
	Best	2.86E + 10	7.76E + 10	4.81E + 10	3.89E + 10	1.03E + 09	4.71E + 10	4.80E + 02	5.26E + 08	1.99E + 02
		5	9	8	6	4	7	2	3	1

*f*_3_	Mean	9.33E + 04	2.12E + 05	9.14E + 04	9.49E + 04	1.08E + 05	9.33E + 04	8.42E + 04	2.39E + 04	1.59E + 04
	Std	1.04E + 03	3.66E + 04	4.26E + 03	1.50E + 04	3.20E + 04	1.43E + 03	2.65E + 03	8.67E + 03	1.06E + 04
	Best	8.89E + 04	1.51E + 05	7.78E + 04	6.06E + 04	5.59E + 04	8.65E + 04	7.76E + 04	8.53E + 03	2.50E + 03
		6	9	4	7	8	5	3	2	1

*f*_4_	Mean	1.22E + 04	4.17E + 04	2.03E + 04	1.45E + 04	1.67E + 03	1.35E + 04	5.86E + 02	1.03E + 03	5.03E + 02
	Std	2.83E + 03	6.73E + 03	6.22E + 02	2.37E + 03	1.06E + 03	1.63E + 03	3.67E + 01	3.67E + 02	2.62E + 01
	Best	7.08E + 03	2.95E + 04	1.90E + 04	8.60E + 03	7.43E + 02	9.85E + 03	5.33E + 02	6.10E + 02	4.00E + 02
		5	9	8	7	4	6	2	3	1

*f*_5_	Mean	8.67E + 02	1.04E + 03	9.34E + 02	9.39E + 02	6.69E + 02	9.67E + 02	7.57E + 02	7.76E + 02	7.05E + 02
	Std	4.03E + 01	4.02E + 01	1.79E + 01	2.49E + 01	4.02E + 01	1.79E + 01	1.16E + 01	3.37E + 01	3.27E + 01
	Best	7.85E + 02	9.46E + 02	8.90E + 02	8.77E + 02	5.94E + 02	9.15E + 02	7.22E + 02	6.92E + 02	6.34E + 02
		5	9	6	7	1	8	3	4	2

*f*_6_	Mean	6.77E + 02	7.12E + 02	6.83E + 02	6.96E + 02	6.22E + 02	6.97E + 02	6.62E + 02	6.55E + 02	6.50E + 02
	Std	7.44E + 00	8.62E + 00	7.87E + 00	6.57E + 00	6.98E + 00	6.82E + 00	2.63E + 00	9.24E + 00	8.74E + 00
	Best	6.58E + 02	6.88E + 02	6.68E + 02	6.70E + 02	6.11E + 02	6.74E + 02	6.56E + 02	6.22E + 02	6.31E + 02
		5	9	6	7	1	8	4	3	2

*f*_7_	Mean	1.43E + 03	3.22E + 03	1.34E + 03	1.43E+03	1.04E+03	1.49E+03	1.01E+03	1.15E+03	9.77E+02
	Std	4.65E + 01	2.45E + 02	2.15E + 01	4.20E + 01	1.54E + 02	2.93E + 01	4.50E + 01	6.49E + 01	4.67E + 01
	Best	1.34E + 03	2.60E + 03	1.29E + 03	1.34E + 03	8.10E + 02	1.42E + 03	9.12E + 02	1.03E + 03	8.94E + 02
		6	9	5	7	3	8	2	4	1

*f*_8_	Mean	1.14E + 03	1.35E + 03	1.13E + 03	1.18E + 03	9.63E + 02	1.17E + 03	9.63E + 02	1.01E + 03	9.77E + 02
	Std	2.40E + 01	3.35E + 01	1.65E + 01	1.54E + 01	3.93E + 01	1.80E + 01	1.098E + 01	2.55E + 01	3.32E + 01
	Best	1.08E + 03	1.28E + 03	1.09E + 03	1.13E + 03	8.97E + 02	1.14E + 03	9.41E + 02	9.50E + 02	9.11E + 02
		6	9	5	8	2	7	1	4	3

*f*_9_	Mean	1.03E + 04	2.66E + 04	1.20E + 04	1.35E + 04	7.47E + 03	1.34E + 04	4.29E + 03	6.55E + 03	3.96E + 03
	Std	1.31E + 03	2.66E + 03	7.66E + 02	1.63E + 03	2.69E + 03	1.48E + 03	3.25E + 02	1.46E + 03	1.76E + 03
	Best	7.40E + 03	2.08E + 04	1.05E + 04	1.05E + 04	3.84E + 03	1.00E + 04	3.67E + 03	3.45E + 03	1.84E + 03
		5	9	6	8	4	7	2	3	1

*f*_10_	Mean	8.98E + 03	9.42E + 03	9.01E + 03	9.09E + 03	5.51E + 03	9.08E + 03	4.30E + 03	6.89E + 03	6.70E + 03
	Std	7.05E + 02	2.94E + 02	2.88E + 02	2.96E + 02	7.47E + 02	4.92E + 02	2.87E + 02	5.29E + 02	7.84E + 02
	Best	7.35E + 03	8.85E + 03	8.16E + 03	7.96E + 03	2.74E + 03	7.27E + 03	3.64E + 03	5.91E + 03	5.01E + 03
		5	9	6	8	2	7	1	3	4

*f*_11_	Mean	1.13E + 04	2.62E + 04	8.41E + 03	1.26E + 04	2.25E + 03	1.29E + 04	4.34E + 03	1.44E + 03	1.27E + 03
	Std	2.60E + 03	7.03E + 03	6.61E + 02	2.83E + 03	1.02E + 03	2.27E + 03	1.00E + 03	1.08E + 02	6.46E + 01
	Best	5.36E + 03	1.32E + 04	7.33E + 03	6.07E + 03	1.35E + 03	8.69E + 03	2.55E + 03	1.27E + 03	1.17E + 03
		6	9	5	7	3	8	4	2	1

*f*_12_	Mean	1.03E + 10	2.57E + 10	2.02E + 10	1.02E + 10	2.14E + 09	1.57E + 10	2.21E + 08	2.15E + 07	6.32E + 05
	Std	3.66E + 09	5.25E + 09	1.75E + 09	1.83E + 09	1.35E + 09	1.90E + 09	1.73E + 08	4.30E + 07	6.09E + 05
	Best	3.64E + 09	1.53E + 10	1.71E + 10	5.28E + 09	6.29E + 06	1.21E + 10	2.48E + 06	4.53E + 05	9.95E + 03
		6	9	8	5	4	7	3	2	1

*f*_13_	Mean	3.13E + 09	1.87E + 10	2.14E + 10	5.36E + 09	5.45E + 08	7.51E + 09	5.52E + 04	3.12E + 04	9.24E + 03
	Std	3.00E + 09	6.86E + 09	5.91E + 09	2.06E + 09	8.21E + 08	2.73E + 09	1.13E + 04	3.05E + 04	6.37E + 03
	Best	2.34E + 08	7.73E + 09	1.11E + 10	1.68E + 09	1.60E + 05	3.86E + 09	2.83E + 04	7.79E + 03	3.46E + 03
		5	8	9	6	4	7	3	2	1

*f*_14_	Mean	8.89E + 06	8.66E + 06	2.44E + 06	3.06E + 06	1.69E + 05	2.35E + 07	1.19E + 06	1.62E + 03	1.62E + 03
	Std	2.80E + 06	5.63E + 06	1.58E + 06	1.64E + 06	1.57E + 05	1.17E + 07	2.04E + 05	7.11E + 01	5.87E + 01
	Best	2.65E + 06	1.39E + 06	4.24E + 05	3.18E + 05	1.12E + 04	3.61E + 06	7.90E + 05	1.50E + 03	1.51E + 03
		8	7	5	6	3	9	4	2	1

*f*_15_	Mean	2.02E + 08	3.26E + 09	6.89E + 08	5.54E + 08	7.47E + 04	8.94E + 08	1.39E + 04	2.52E + 03	2.02E + 03
	Std	2.42E + 08	1.43E + 09	1.98E + 08	2.05E + 08	5.55E + 04	2.83E + 08	2.16E + 03	7.65E + 02	1.79E + 02
	Best	6.13E + 05	8.12E + 08	1.65E + 08	6.43E + 07	1.29E + 04	6.51E + 08	8.68E + 03	1.87E + 03	1.76E + 03
		5	9	7	6	4	8	3	2	1

*f*_16_	Mean	5.30E + 03	6.80E + 03	9.95E + 03	5.35E + 03	2.93E + 03	6.32E + 03	3.68E + 03	3.25E + 03	2.98E + 03
	Std	6.47E + 02	9.04E + 02	4.55E + 02	3.90E + 02	4.42E + 02	7.14E + 02	2.10E + 02	3.68E + 02	2.52E + 02
	Best	3.55E + 03	5.13E + 03	9.23E + 03	4.24E + 03	2.02E + 03	5.23E + 03	3.26E + 03	2.50E + 03	2.30E + 03
		5	8	9	6	1	7	4	3	2

*f*_17_	Mean	4.41E + 03	8.30E + 03	1.79E + 04	3.68E + 03	2.40E + 03	2.84E + 04	2.87E + 03	2.15E + 03	2.13E + 03
	Std	4.01E + 03	6.47E + 03	2.23E + 03	2.50E + 02	2.58E + 02	1.63E + 04	1.99E + 02	1.84E + 02	1.31E + 02
	Best	2.38E + 03	3.13E + 03	1.40E + 04	3.21E + 03	2.03E + 03	5.49E + 03	2.52E + 03	1.82E + 03	1.86E + 03
		6	7	8	5	3	9	4	2	1

*f*_18_	Mean	1.53E + 08	1.71E + 08	9.62E + 07	3.88E + 07	5.07E + 06	2.76E + 08	1.76E + 06	2.72E + 03	3.77E + 03
	Std	6.49E + 07	1.28E + 08	3.16E + 07	2.03E + 07	3.53E + 06	1.39E + 08	4.33E + 05	9.95E + 02	5.78E + 03
	Best	7.80E + 06	7.75E + 06	2.23E + 07	6.27E + 06	1.80E + 05	1.17E + 07	8.06E + 05	2.06E + 03	2.23E + 03
		7	8	6	5	4	9	3	1	2

*f*_19_	Mean	4.82E + 08	4.34E + 09	1.47E + 09	6.72E + 08	1.19E + 07	8.44E + 08	2.54E + 04	2.51E + 03	2.04E + 03
	Std	3.82E + 08	2.23E + 09	4.66E + 08	2.41E + 08	3.16E + 07	5.06E + 08	1.01E + 04	2.13E + 03	5.89E + 01
	Best	6.66E + 06	5.38E + 08	2.09E + 08	2.92E + 08	2.16E + 03	2.14E + 08	1.20E + 04	1.97E + 03	1.96E + 03
		5	9	8	6	4	7	3	2	1

*f*_20_	Mean	3.02E + 03	3.29E + 03	3.11E + 03	3.11E + 03	2.52E + 03	3.28E + 03	3.43E + 03	2.53E + 03	2.49E + 03
	Std	2.13E + 02	1.57E + 02	1.16E + 02	1.22E + 02	1.78E + 02	1.79E + 02	1.43E + 02	1.17E + 02	8.70E + 01
	Best	2.45E + 03	2.92E + 03	2.65E + 03	2.67E + 03	2.14E + 03	2.90E + 03	3.11E + 03	2.26E + 03	2.30E + 03
		4	8	5	6	3	7	9	2	1

*f*_21_	Mean	2.71E + 03	2.87E + 03	2.75E + 03	2.74E + 03	2.45E + 03	2.78E + 03	2.61E + 03	2.51E + 03	2.49E + 03
	Std	3.46E + 01	4.51E + 01	1.63E + 01	2.79E + 01	3.88E + 01	3.94E + 01	2.16E + 01	4.08E + 01	3.35E + 01
	Best	2.65E + 03	2.76E + 03	2.71E + 03	2.67E + 03	2.39E + 03	2.68E + 03	2.57E + 03	2.44E + 03	2.43E + 03
		5	9	7	6	1	8	4	3	2

*f*_22_	Mean	8.22E + 03	1.06E + 04	6.38E + 03	8.87E + 03	6.87E + 03	1.02E + 04	7.35E + 03	3.37E + 03	2.30E + 03
	Std	8.42E + 02	6.88E + 02	3.04E + 02	7.69E + 02	8.79E + 02	4.47E + 02	2.65E + 02	6.38E + 02	3.51E + 00
	Best	6.50E + 03	8.60E + 03	5.64E + 03	7.00E + 03	5.06E + 03	9.06E + 03	6.86E + 03	2.67E + 03	2.30E + 03
		6	9	3	7	4	8	5	2	1

*f*_23_	Mean	3.50E + 03	3.62E + 03	3.60E + 03	3.40E + 03	2.97E + 03	3.70E + 03	3.77E + 03	2.97E + 03	2.96E + 03
	Std	8.07E + 01	1.06E + 02	5.72E + 01	8.23E + 01	6.53E + 01	1.40E + 02	1.38E + 02	6.95E + 01	6.43E + 01
	Best	3.36E + 03	3.35E + 03	3.50E + 03	3.17E + 03	2.85E + 03	3.39E + 03	3.54E + 03	2.84E + 03	2.83E + 03
		5	7	6	4	3	8	9	2	1

*f*_24_	Mean	4.36E + 03	4.03E + 03	4.28E + 03	3.59E + 03	3.18E + 03	3.97E + 03	3.38E + 03	3.16E + 03	3.12E + 03
	Std	2.56E + 02	2.48E + 02	6.10E + 01	9.75E + 01	5.83E + 01	8.84E + 01	6.03E + 01	7.98E + 01	9.18E + 01
	Best	3.75E + 03	3.46E + 03	4.12E + 03	3.42E + 03	3.01E + 03	3.76E + 03	3.24E + 03	3.01E + 03	2.99E + 03
		9	7	8	5	3	6	4	2	1

*f*_25_	Mean	4.48E + 03	1.64E + 04	6.44E + 03	5.31E + 03	3.49E + 03	4.96E + 03	2.98E + 03	3.19E + 03	2.90E + 03
	Std	3.21E + 02	2.74E + 03	1.35E + 02	3.66E + 02	4.81E + 02	3.89E + 02	1.24E + 01	1.26E + 02	1.63E + 01
	Best	3.89E + 03	1.14E + 04	6.13E + 03	4.25E + 03	2.94E + 03	4.13E + 03	2.95E + 03	3.02E + 03	2.88E + 03
		5	9	8	7	4	6	2	3	1

*f*_26_	Mean	1.18E + 04	1.52E + 04	1.04E + 04	1.09E + 04	5.44E + 03	1.23E + 04	7.81E + 03	6.83E + 03	5.26E + 03
	Std	1.10E + 03	1.78E + 03	1.55E + 02	6.27E + 02	9.26E + 02	8.66E + 02	3.71E + 02	8.40E + 02	1.25E + 03
	Best	9.62E + 03	1.06E + 04	9.75E + 03	8.93E + 03	4.16E + 03	1.06E + 04	7.15E + 03	4.22E + 03	2.81E + 03
		7	9	5	6	2	8	4	3	1

*f*_27_	Mean	4.23E + 03	4.66E + 03	4.12E + 03	4.08E + 03	3.31E + 03	4.81E + 03	5.02E + 03	3.28E + 03	3.26E + 03
	Std	1.55E + 02	3.19E + 02	1.63E + 02	1.62E + 02	4.57E + 01	6.22E + 02	1.74E + 02	4.52E + 01	4.85E + 01
	Best	3.86E + 03	3.87E + 03	3.83E + 03	3.71E + 03	3.24E + 03	4.10E + 03	4.45E + 03	3.21E + 03	3.19E + 03
		6	7	5	4	3	8	9	2	1

*f*_28_	Mean	6.64E + 03	1.10E + 04	8.53E + 03	7.08E + 03	5.98E + 03	6.87E + 03	3.51E + 03	3.78E + 03	3.23E + 03
	Std	7.57E + 02	1.33E + 03	9.24E + 01	4.71E + 02	1.19E + 03	5.67E + 02	1.49E + 02	2.27E + 02	2.62E + 01
	Best	5.00E + 03	7.96E + 03	8.30E + 03	6.06E + 03	3.51E + 03	5.72E + 03	3.36E + 03	3.42E + 03	3.20E + 03
		5	9	8	7	4	6	2	3	1

*f*_29_	Mean	5.91E + 03	8.88E + 03	1.81E + 04	6.51E + 03	4.14E + 03	8.08E + 03	4.93E + 03	4.26E + 03	4.05E + 03
	Std	5.43E + 02	3.35E + 03	2.44E + 03	4.38E + 02	2.95E + 02	1.28E + 03	2.35E + 02	2.60E + 02	2.17E + 02
	Best	4.87E + 03	5.72E + 03	1.24E + 04	5.48E + 03	3.68E + 03	6.22E + 03	4.50E + 03	3.59E + 03	3.66E + 03
		5	8	9	6	2	7	4	3	1

*f*_30_	Mean	1.85E + 08	1.28E + 09	3.01E + 09	6.99E + 08	1.28E + 07	2.75E + 09	9.00E + 05	2.48E + 05	3.07E + 04
	Std	9.84E + 07	3.65E + 08	1.46E + 09	2.42E + 08	1.50E + 07	9.75E + 07	4.35E + 05	1.30E + 06	2.07E + 04
	Best	5.82E + 07	3.33E + 08	8.00E + 08	1.93E + 08	2.94E + 04	2.29E + 09	1.26E + 05	9.35E + 03	7.49E + 03
		5	7	9	6	4	8	3	2	1

Total rank		163	244	192	181	92	214	106	74	39
Final rank		5	9	7	6	3	8	4	2	1

**Table 5:** Experimental results of FCBAISA and other algorithms in CEC2022 benchmark functions.

Function		TSO	AHO	BOA	DDAO20	PSO	OSA	GSA	AISA	FCBAISA
*f*_1_	Mean	5.32E + 04	5.95E + 04	5.11E + 04	4.29E + 04	1.81E + 04	9.45E + 04	2.42E + 04	5.59E + 02	**4.85E** + **02**
	Std	3.58E + 04	9.11E + 03	1.66E + 04	7.39E + 03	1.56E + 04	3.95E + 04	4.60E + 03	2.83E + 02	**2.23E** + **02**
	Best	2.00E + 04	3.48E + 04	2.48E + 04	2.57E + 04	3.09E + 02	3.11E + 04	1.59E + 04	3.21E + 02	**3.01E** + **02**
		7	8	6	5	3	9	4	2	**1**

*f*_2_	Mean	1.90E + 03	5.48E + 03	3.66E + 03	2.02E + 03	5.86E + 02	3.12E + 03	4.68E + 02	5.57E + 02	**4.47E** + **02**
	Std	5.89E + 02	1.64E + 03	7.47E + 02	2.44E + 02	1.14E + 02	7.16E + 02	1.96E + 01	6.14E + 01	**1.02E** + **01**
	Best	9.08E + 02	2.10E + 03	2.33E + 03	1.30E + 03	4.46E + 02	2.05E + 03	4.01E + 02	4.69E + 02	4.07E + 02
		5	9	8	6	4	7	2	3	**1**

*f*_3_	Mean	6.73E + 02	6.92E + 02	6.67E + 02	6.75E + 02	6.15E + 02	6.87E + 02	6.36E + 02	6.34E + 02	**6.04E** + **02**
	Std	1.15E + 01	1.04E + 01	1.13E + 01	5.62E + 00	5.96E + 00	9.85E + 00	9.58E + 00	1.08E + 01	1.64E + 00
	Best	6.50E + 02	6.69E + 02	6.34E + 02	6.63E + 02	6.04E + 02	6.65E + 02	6.07E + 02	6.12E + 02	**6.01E** + **02**
		6	9	5	7	2	8	4	3	**1**

*f*_4_	Mean	9.58E + 02	1.05E + 03	9.68E + 02	9.83E + 02	8.73E + 02	9.80E + 02	8.75E + 02	8.75E + 02	**8.51E** + **02**
	Std	1.74E + 01	1.82E + 01	1.02E + 01	1.07E + 01	2.31E + 01	1.56E + 01	1.06E + 01	1.40E + 01	1.47E + 01
	Best	9.22E + 02	1.01E + 03	9.44E + 02	9.40E + 02	8.36E + 02	9.38E + 02	8.51E + 02	8.37E + 02	**8.22E** + **02**
		5	9	6	8	2	7	4	3	**1**

*f*_5_	Mean	3.25E + 03	8.40E + 03	3.24E + 03	3.96E + 03	1.75E + 03	3.81E + 03	**9.64E** + **02**	1.82E + 03	9.79E + 02
	Std	3.95E + 02	1.15E + 03	3.46E + 02	4.39E + 02	6.04E + 02	4.03E + 02	**1.16E** + **02**	4.29E + 02	5.29E + 01
	Best	2.43E + 03	6.38E + 03	2.25E + 03	2.95E + 03	9.02E + 02	2.97E + 03	**9.00E** + **02**	1.11E + 03	9.08E + 02
		6	9	5	8	3	7	**1**	4	2

*f*_6_	Mean	1.16E + 09	3.51E + 09	2.62E + 09	1.08E + 09	1.79E + 07	3.40E + 09	3.06E + 03	1.96E + 03	**1.89E** + **03**
	Std	1.08E + 09	1.30E + 09	1.29E + 09	3.55E + 08	1.91E + 07	1.14E + 09	1.22E + 03	6.52E + 01	**4.50E** + **01**
	Best	8.62E + 06	6.99E + 08	1.91E + 08	3.33E + 08	2.13E + 03	1.33E + 09	1.95E + 03	1.87E + 03	**1.82E** + **03**
		6	9	7	5	4	8	3	2	**1**

*f*_7_	Mean	2.20E + 03	2.26E + 03	2.17E + 03	2.19E + 03	2.08E + 03	2.25E + 03	2.36E + 03	2.08E + 03	**2.05E** + **03**
	Std	3.63E + 01	4.42E + 01	2.47E + 01	2.54E + 01	3.96E + 01	5.84E + 01	6.65E + 01	2.62E + 01	**1.34E** + **01**
	Best	2.11E + 03	2.17E + 03	2.12E + 03	2.13E + 03	2.03E + 03	2.17E + 03	2.20E + 03	2.03E + 03	**2.03E** + **03**
		6	8	4	5	3	7	9	2	**1**

*f*_8_	Mean	2.30E + 03	2.69E + 03	5.42E + 03	2.41E + 03	2.27E + 03	2.34E + 03	2.51E + 03	2.23E + 03	**2.23E** + **03**
	Std	9.10E + 01	2.81E + 02	6.76E + 03	8.65E + 01	6.12E + 01	1.13E + 02	1.06E + 02	3.28E + 00	**2.21E** + **00**
	Best	2.23E + 03	2.26E + 03	2.33E + 03	2.27E + 03	2.22E + 03	2.24E + 03	2.23E + 03	2.22E + 03	2.23E + 03
		4	8	9	6	3	5	7	2	**1**

*f*_9_	Mean	2.96E + 03	3.23E + 03	3.99E + 03	2.87E + 03	2.59E + 03	3.73E + 03	2.51E + 03	2.49E + 03	**2.48E** + **03**
	Std	1.99E + 02	1.85E + 02	5.33E + 02	8.58E + 01	9.58E + 01	3.09E + 02	1.60E + 01	1.03E + 01	**2.14E-01**
	Best	2.65E + 03	2.88E + 03	3.05E + 03	2.70E + 03	2.49E + 03	3.10E + 03	2.49E + 03	2.48E + 03	2.48E + 03
		6	7	9	5	4	8	3	2	**1**

*f*_10_	Mean	5.82E + 03	6.21E + 03	3.23E + 03	2.80E + 03	4.08E + 03	6.56E + 03	4.75E + 03	2.90E + 03	**2.66E** + **03**
	Std	1.29E + 03	1.36E + 03	1.44E + 03	2.07E + 02	9.76E + 02	7.91E + 02	6.23E + 02	5.75E + 02	**5.58E** + **02**
	Best	2.58E + 03	2.69E + 03	2.52E + 03	2.55E + 03	2.52E + 03	3.29E + 03	2.50E + 03	2.50E + 03	2.50E + 03
		7	8	4	2	5	9	6	3	**1**

*f*_11_	Mean	7.73E + 03	1.08E + 04	9.05E + 03	7.14E + 03	5.05E + 03	9.04E + 03	**2.91E** + **03**	3.84E + 03	3.01E + 03
	Std	1.13E + 03	1.43E + 03	5.81E + 02	7.14E + 02	1.01E + 03	5.09E + 02	**1.05E** + **02**	4.94E + 02	1.05E + 02
	Best	4.83E + 03	7.04E + 03	7.13E + 03	5.03E + 03	3.34E + 03	7.87E + 03	**2.60E** + **03**	3.10E + 03	2.82E + 03
		6	9	8	5	4	7	**1**	3	2

*f*_12_	Mean	3.56E + 03	3.64E + 03	3.30E + 03	3.41E + 03	3.03E + 03	4.36E + 03	3.71E + 03	3.01E + 03	**2.94E** + **03**
	Std	3.28E + 02	1.70E + 02	1.09E + 02	7.92E + 01	6.48E + 01	3.84E + 02	2.46E + 02	5.77E + 01	**3.73E** + **00**
	Best	3.06E + 03	3.25E + 03	3.09E + 03	3.22E + 03	2.95E + 03	3.55E + 03	3.15E + 03	2.95E + 03	**2.93E** + **03**
		6	7	4	5	3	9	8	2	**1**

Total rank		70	100	75	67	40	91	52	31	**14**
Final rank		6	9	7	5	3	8	4	2	**1**

**Table 6:** The results of Wilcoxon rank test for the benchmark functions of CEC2017.

Function	FCBAISA vs AISA				FCBAISA vs PSO				FCBAISA vs BOA				FCBAISA vs DDAO			
	*p*_value	*R* ^+^	*R* ^−^	+/=/−	*p*_value	*R* ^+^	*R* ^−^	+/=/−	*p*_value	*R* ^+^	*R* ^−^	+/=/−	*p*_value	*R* ^+^	*R* ^−^	+/=/−
*f*_1_	7.56E-10	0	1257	+	7.56E-10	0	1275	+	7.55E-10	0	1230	+	7.56E-10	0	1275	+
*f*_3_	6.42E-04	284	766	+	7.55E-10	0	1274	+	7.56E-10	0	1275	+	7.55E-10	0	1274	+
*f*_4_	7.50E-10	0	1200	+	7.56E-10	0	1173	+	7.55E-10	0	1184	+	7.55E-10	0	1275	+
*f*_5_	1.85E-09	15	1095	+	7.71E-05	1047	180	−	7.55E-10	0	1260	+	7.56E-10	0	1275	+
*f*_6_	4.30E-03	342	758	+	7.55E-10	1253	0	−	8.03E-10	1	1258	+	7.56E-10	0	1273	+
*f*_7_	7.50E-10	0	1230	+	9.80E-03	370	905	+	7.55E-10	0	1258	+	7.55E-10	0	1272	+
*f*_8_	2.88E-07	80	1169	+	7.18E-02	777	430	=	7.55E-10	0	1268	+	7.54E-10	0	1270	+
*f*_9_	3.43E-08	66	1014	+	1.07E-07	87	1142	+	7.55E-10	0	1186	+	7.53E-10	0	1271	+
*f*_10_	4.37E-01	618	557	=	4.51E-09	1182	26	−	7.54E-10	0	1240	+	8.03E-10	1	1274	+
*f*_11_	4.76E-09	31	1158	+	7.55E-10	0	1234	+	7.55E-10	0	1233	+	7.55E-10	0	1270	+
*f*_12_	8.01E-10	1	1162	+	7.55E-10	0	1258	+	7.55E-10	0	1268	+	7.56E-10	0	1272	+
*f*_13_	7.77E-08	81	1054	+	7.55E-10	0	1275	+	7.55E-10	0	1235	+	7.55E-10	0	1271	+
*f*_14_	9.96E-01	637	582	=	7.55E-10	0	1230	+	7.55E-10	0	1212	+	7.56E-10	0	1270	+
*f*_15_	8.35E-06	96	1099	+	7.56E-10	0	1275	+	7.55E-10	0	1226	+	7.56E-10	0	1270	+
*f*_16_	1.02E-04	235	835	+	3.88E-01	706	509	=	7.55E-10	0	1194	+	7.56E-10	0	1270	+
*f*_17_	1.54E-01	460	785	=	6.99E-08	79	1145	+	7.55E-10	0	1217	+	7.55E-10	0	1270	+
*f*_18_	6.00E-03	860	353	−	7.56E-10	0	1240	+	7.55E-10	0	1173	+	7.55E-10	0	1270	+
*f*_19_	9.78E-05	234	836	+	7.55E-10	0	1232	+	7.55E-10	0	1250	+	7.55E-10	0	1270	+
*f*_20_	3.57E-01	530	733	=	2.04E-01	506	769	=	7.56E-10	0	1275	+	7.55E-10	0	1270	+
*f*_21_	4.30E-03	297	894	+	4.53E-05	1060	215	−	7.54E-10	0	1179	+	7.55E-10	0	1270	+
*f*_22_	7.54E-10	0	1227	+	7.56E-10	0	1226	+	7.56E-10	0	1226	+	7.56E-10	0	1270	+
*f*_23_	9.50E-01	631	609	=	3.82E-01	547	716	=	7.55E-10	0	1225	+	7.56E-10	0	1275	+
*f*_24_	2.67E-02	213	867	+	1.63E-04	170	1028	+	7.55E-10	0	1260	+	7.56E-10	0	1273	+
*f*_25_	7.50E-10	0	1225	+	7.55E-10	0	1228	+	7.55E-10	0	1214	+	7.56E-10	0	1274	+
*f*_26_	5.91E-08	76	1034	+	9.96E-01	553	638	=	7.56E-10	0	1275	+	7.56E-10	0	1271	+
*f*_27_	1.36E-01	483	703	=	6.44E-04	284	981	+	7.56E-10	0	1233	+	7.56E-10	0	1273	+
*f*_28_	7.52E-10	0	1209	+	7.56E-10	0	1275	+	7.55E-10	0	1182	+	7.56E-10	0	1268	+
*f*_29_	2.20E-05	198	1011	+	7.81E-02	455	770	=	7.54E-10	0	1101	+	7.56E-10	0	1275	+
*f*_30_	2.50E-03	324	796	+	7.55E-10	0	1192	+	7.56E-10	0	1245	+	7.56E-10	0	1275	+
Total				22/6/1				19/6/4				29/0/0				29/0/0

**Table 7:** The results of Wilcoxon rank test for the benchmark functions of CEC2017.

Function	FCBAISA vs AHO				FCBAISA vs OSA				FCBAISA vs TSO				FCBAISA vs GSA			
	*p*_value	*R* ^+^	*R* ^−^	+/=/−	*p*_value	*R* ^+^	*R* ^−^	+/=/−	*p*_value	*R* ^+^	*R* ^−^	+/=/−	*p*_value	*R* ^+^	*R* ^−^	+/=/−
*f*_1_	7.55E-10	0	1270	+	7.55E-10	0	1221	+	7.56E-10	0	1270	+	3.82E-01	547	728	=
*f*_3_	7.52E-10	0	1232	+	7.55E-10	0	1180	+	7.56E-10	0	1270	+	7.55E-10	0	1178	+
*f*_4_	7.50E-10	0	1270	+	7.54E-10	0	1161	+	7.55E-10	0	1270	+	8.01E-10	1	1172	+
*f*_5_	7.53E-10	0	1270	+	7.55E-10	0	1168	+	7.55E-10	0	1270	+	1.30E-09	9	1188	+
*f*_6_	7.55E-10	0	1270	+	7.55E-10	0	1228	+	8.03E-10	1	1274	+	1.26E-08	48	1162	+
*f*_7_	7.52E-10	3	1268	+	7.54E-10	0	1230	+	7.54E-10	5	1162	+	1.50E-03	267	966	+
*f*_8_	7.53E-10	7	1270	+	7.55E-10	0	1267	+	7.55E-10	0	1275	+	9.30E-03	760	368	−
*f*_9_	7.48E-10	0	1275	+	7.55E-10	0	1132	+	7.46E-10	0	1252	+	5.41E-02	438	792	=
*f*_10_	7.32E-10	0	1275	+	7.55E-10	0	1204	+	9.07E-10	3	1272	+	7.55E-10	1222	0	−
*f*_11_	7.43E-10	9	1275	+	7.54E-10	0	1143	+	7.49E-10	0	1260	+	7.38E-10	0	1152	+
*f*_12_	7.81E-10	0	1237	+	7.55E-10	0	1196	+	6.58E-10	0	1255	+	7.39E-10	0	1128	+
*f*_13_	7.42E-10	2	1270	+	7.55E-10	0	1197	+	7.51E-10	0	1267	+	7.53E-10	0	1209	+
*f*_14_	7.49E-10	0	1271	+	7.55E-10	0	1226	+	7.53E-10	0	1273	+	7.51E-10	0	1194	+
*f*_15_	7.45E-10	0	1238	+	7.55E-10	0	1203	+	7.54E-10	0	1245	+	7.55E-10	0	1266	+
*f*_16_	7.48E-10	0	1235	+	7.55E-10	0	1243	+	7.48E-10	0	1236	+	7.57E-10	0	1161	+
*f*_17_	7.37E-10	0	1272	+	7.55E-10	0	1156	+	7.49E-10	0	1269	+	7.50E-10	0	1266	+
*f*_18_	7.51E-10	0	1269	+	7.54E-10	0	1053	+	7.48E-10	0	1270	+	7.49E-10	0	1170	+
*f*_19_	7.47E-10	0	1272	+	7.52E-10	0	1217	+	7.52E-10	0	1270	+	7.53E-10	0	1227	+
*f*_20_	7.52E-10	0	1262	+	7.55E-10	0	1180	+	8.03E-10	1	1274	+	7.55E-10	0	1238	+
*f*_21_	7.43E-10	0	1254	+	7.55E-10	0	1145	+	7.46E-10	0	1267	+	7.54E-10	0	1167	+
*f*_22_	7.49E-10	0	1270	+	7.55E-10	0	1204	+	7.43E-10	0	1252	+	7.55E-10	0	1206	+
*f*_23_	7.39E-10	0	1273	+	7.54E-10	0	1173	+	7.48E-10	0	1243	+	7.54E-10	0	1131	+
*f*_24_	7.51E-10	0	1270	+	7.55E-10	0	1268	+	7.50E-10	0	1241	+	1.38E-09	4	1265	+
*f*_25_	7.52E-10	0	1265	+	7.55E-10	0	1225	+	7.39E-10	4	1270	+	7.55E-10	0	1216	+
*f*_26_	7.44E-10	0	1267	+	7.55E-10	0	1190	+	7.48E-10	0	1269	+	7.55E-10	0	1202	+
*f*_27_	7.50E-10	0	1267	+	7.55E-10	0	1210	+	7.52E-10	0	1263	+	7.54E-10	0	1167	+
*f*_28_	7.41E-10	0	1251	+	7.52E-10	0	1133	+	7.53E-10	0	1268	+	7.54E-10	0	1173	+
*f*_29_	7.47E-10	0	1235	+	7.52E-10	0	1239	+	7.51E-10	1	1270	+	8.01E-10	1	1193	+
*f*_30_	7.52E-10	3	1272	+	7.54E-10	0	1122	+	7.52E-10	2	1273	+	7.54E-10	0	1140	+
Total				29/0/0				29/0/0				29/0/0				25/2/2

**Table 8:** The results of Friedman test for the benchmark functions of CEC2017.

Name	Score	Rank
AHO	1.5517	9
OSA	2.6724	8
BOA	3.3621	7
DDAO	3.7759	6
TSO	4.3620	5
GSA	6.3793	4
PSO	6.8448	3
AISA	7.3965	2
FCBAISA	**8.6552**	**1**
*p*_value	**1.2942E-10**	

**Table 9:** The results of Quade test for the benchmark functions of CEC2017.

Name	Score	Rank
AHO	1.5264	9
OSA	2.7701	8
BOA	3.0414	7
DDAO	3.8103	6
TSO	4.3229	5
PSO	6.3506	4
GSA	6.7701	3
AISA	7.5851	2
FCBAISA	**8.8229**	**1**
*p*_value	**2.2054E-43**	

**Table 10:** Experimental results of pressure vessel design problem.

	TSO	AHO	BOA	DDAO	PSO	OSA	GSA	AISA	FCBAISA
*x*_1_	0.9129	0.8948	1.139	0.8572	0.7851	0.7859	0.9297	0.7866	0.7824
*x*_2_	0.4688	0.4708	0.5321	0.481	0.4064	0.4724	0.4822	0.4063	0.4076
*x*_3_	46.6098	40.6415	53.8072	42.2844	40.3196	40.9759	47.5163	42.0984	42.0909
*x*_4_	11.4509	12.8559	10.0000	10.0000	10.0000	45.7413	56.4759	62.7144	140.3024
Min	9.98E + 03	1.071E + 04	1.05E + 04	9.42E + 03	6.45E + 03	9.39E + 03	1.07E + 04	6.14E + 03	**6.06E** + **03**

**Table 11:** Experimental results of welded beam design problem.

	TSO	AHO	BOA	DDAO	PSO	OSA	GSA	AISA	FCBAISA
*x*_1_	0.1544	0.1501	0.2578	0.1557	0.166	0.1518	0.2088	0.1658	0.1659
*x*_2_	3.3508	2.6955	3.3616	3.7372	8.2326	3.0559	3.8553	7.4115	8.2326
*x*_3_	4.9116	6.8373	5.7222	7.0623	9.9971	4.3309	5.7837	9.9936	9.9971
*x*_4_	0.1733	0.1687	0.2518	0.1841	0.168	0.1696	0.1879	0.168	0.168
Min	2.9727	2.8871	3.337	2.8895	2.0632	2.677	2.8778	2.0638	**2.0632**

**Table 12:** Experimental results of gear train design problem.

	TSO	AHO	BOA	DDAO	PSO	OSA	GSA	AISA	FCBAISA
*x*_1_	12.0222	12.0000	12.0000	12.37856	12.0000	12.0000	13.2554	12.0000	12.0000
*x*_2_	12.8303	12.0000	12.0000	12.0000	12.0000	20.0455	12.2594	12.0693	12.3333
*x*_3_	51.9823	19.03763	24.0755	29.2456	23.1030	35.1536	43.7907	23.0162	34.3642
*x*_4_	12.8303	20.97001	21.6255	24.4759	18.0335	31.4768	43.5781	23.3093	34.1505
Min	0.0020	3.20E-08	0.0178	6.40E-08	4.14E-08	3.57E-02	6.81E-10	3.09E-10	**2.23E-10**

**Table 13:** Experimental results of speed reducer design problem.

	TSO	AHO	BOA	DDAO	PSO	OSA	GSA	AISA	FCBAISA
*x*_1_	2.6025	3.5012	2.7400	3.5039	2.6000	2.7698	3.5177	2.9963	3.5000
*x*_2_	0.7000	0.7000	0.7000	0.7000	0.7000	0.7878	0.7006	0.7000	0.7000
*x*_3_	17.0000	17.0000	17.0000	17.0000	17.0000	18.0818	17.0330	17.0000	17.0000
*x*_4_	7.3000	7.3000	7.3000	7.3000	7.3000	7.7211	7.3282	7.3000	7.3000
*x*_5_	7.8000	7.8000	7.8000	7.8139	7.8000	8.2789	7.8080	7.8000	7.8000
*x*_6_	3.3492	3.355	3.3543	3.3517	3.3486	8.4332	3.3528	3.3497	3.3502
*x*_7_	5.2864	5.2895	5	5.2891	5.2862	5.3459	5.2947	5.2864	5.2865
Min	7.63E + 05	3.14E + 03	9.43E + 05	3.31E + 03	4.03E + 05	1.00E + 06	3.53E + 03	6.30E + 04	**3.00E** + **03**

## Data Availability

The data used to support the findings of this study are included within the article.
